# Multi-Modal Machine Learning Approach for COVID-19 Detection Using Biomarkers and X-Ray Imaging

**DOI:** 10.3390/diagnostics14242800

**Published:** 2024-12-13

**Authors:** Kagan Tur

**Affiliations:** Internal Medicine Department, Faculty of Medicine, Ahi Evran University, Kirsehir 40200, Turkey; kagan.tur@ahievran.edu.tr; Tel.: +90-50-577-339-27

**Keywords:** multi-modal machine learning, COVID-19 diagnostics, biomarkers and imaging, SHAP and LIME analysis

## Abstract

*Background*: Accurate and rapid detection of COVID-19 remains critical for clinical management, especially in resource-limited settings. Current diagnostic methods face challenges in terms of speed and reliability, creating a need for complementary AI-based models that integrate diverse data sources. *Objectives*: This study aimed to develop and evaluate a multi-modal machine learning model that combines clinical biomarkers and chest X-ray images to enhance diagnostic accuracy and provide interpretable insights. *Methods*: We used a dataset of 250 patients (180 COVID-19 positive and 70 negative cases) collected from clinical settings. Biomarkers such as CRP, ferritin, NLR, and albumin were included alongside chest X-ray images. Random Forest and Gradient Boosting models were used for biomarkers, and ResNet and VGG CNN architectures were applied to imaging data. A late-fusion strategy integrated predictions from these modalities. Stratified k-fold cross-validation ensured robust evaluation while preventing data leakage. Model performance was assessed using AUC-ROC, F1-score, Specificity, Negative Predictive Value (NPV), and Matthews Correlation Coefficient (MCC), with confidence intervals calculated via bootstrap resampling. *Results*: The Gradient Boosting + VGG fusion model achieved the highest performance, with an AUC-ROC of 0.94, F1-score of 0.93, Specificity of 93%, NPV of 96%, and MCC of 0.91. SHAP and LIME interpretability analyses identified CRP, ferritin, and specific lung regions as key contributors to predictions. *Discussion*: The proposed multi-modal approach significantly enhances diagnostic accuracy compared to single-modality models. Its interpretability aligns with clinical understanding, supporting its potential for real-world application.

## 1. Introduction

COVID-19 continues to challenge global healthcare systems, underscoring the need for rapid, accurate, and scalable diagnostic methods. While RT-PCR remains the gold standard for COVID-19 detection, it is often hampered by high false-negative rates, slow processing times, and limited accessibility in resource-constrained environments [1,2,3]. These limitations have prompted growing interest in complementary diagnostic approaches that integrate advanced technologies, such as machine learning and artificial intelligence (AI), to analyze clinical and imaging data [4,5].

Clinical biomarkers such as C-reactive protein (CRP), ferritin, and Neutrophil-to-Lymphocyte Ratio (NLR) provide systemic insights into inflammation and immune responses, while chest X-ray images visualize respiratory pathology, including ground-glass opacities and consolidations commonly observed in COVID-19 patients [6,7,8]. Integrating these data sources has the potential to significantly enhance diagnostic accuracy. Recent studies have shown that chest X-rays are particularly valuable for identifying COVID-19-related pneumonia and distinguishing it from other respiratory conditions, yet these approaches often lack the systemic context provided by biomarkers [9,10].

Machine learning has been widely applied to COVID-19 diagnostics, but most studies focus on a single data modality—either imaging or biomarkers—leaving a gap in utilizing their combined diagnostic potential. For example, deep learning architectures like ResNet and VGG have demonstrated high AUC-ROC scores (>0.9) in analysing chest X-ray data, making them effective tools for detecting COVID-19-related lung abnormalities. However, these models often lack insights into systemic inflammation and immune responses that can be captured through biomarkers [11,12]. Similarly, biomarker-based models excel in identifying systemic features of COVID-19 but lack the detailed imaging insights critical for understanding localized lung pathology [13]. Recent research, such as COVID-Net, has explored interpretability in imaging but focuses exclusively on X-ray data, neglecting the systemic context that biomarkers could provide [14]. This represents a significant gap in leveraging the complementary strengths of multi-modal data.

Transparency and interpretability are essential for the adoption of machine learning models in clinical settings. However, most existing studies lack robust interpretability frameworks, limiting their clinical applicability. While some imaging-focused studies use Grad-CAM or saliency maps to highlight regions of interest, these approaches are largely confined to single-modality datasets, failing to generalize across multi-modal data combinations [15,16]. Few studies incorporate advanced interpretable techniques like SHAP or LIME to provide a detailed understanding of how models arrive at predictions, leaving a critical gap in aligning machine learning outputs with clinical reasoning [17].

This study addresses these critical gaps by developing a multi-modal machine learning framework that integrates systemic biomarkers with imaging data for COVID-19 detection. The novelty of this approach lies in three main contributions.

First, the study leverages synergistic integration, combining systemic biomarkers such as CRP, ferritin, and NLR with imaging features extracted from chest X-rays. This integration enhances diagnostic accuracy by combining localized and systemic disease information, bridging the gap left by single-modality models. Second, it employs state-of-the-art interpretability techniques, including SHAP and LIME, to ensure that the model predictions align with clinical reasoning and physiological mechanisms. This interpretability adds a critical layer of transparency and trust, enabling clinicians to better understand and utilize the model predictions. Finally, this study addresses dataset imbalance by utilizing stratified k-fold cross-validation, ensuring fair and unbiased performance evaluation across COVID-19-positive and negative cases.

The proposed multi-modal framework represents a significant advancement over traditional approaches, offering a robust, interpretable, and clinically applicable tool for improving COVID-19 diagnostics.

## 2. Materials and Methods

The dataset used in this study included a diverse sample of 250 cases, comprising 180 COVID-19 positive cases and 70 COVID-19 negative cases, providing a robust basis for evaluating model performance. Patient data comprised demographic information, blood biomarkers, and chest X-ray images, enabling a comprehensive multi-modal analysis. The dataset included patients from Middle and Greater Anatolia, ensuring demographic diversity in terms of age, gender, and geographic distribution. This inclusion enhances the generalizability of the findings and aligns the study with the clinical demographics encountered in regional healthcare settings.

Key biomarkers analyzed included C-reactive protein (CRP), ferritin, the Neutrophil-to-Lymphocyte Ratio (NLR), the Monocyte-to-Lymphocyte Ratio (MLR) and albumin [18]. These biomarkers were selected for their established associations with COVID-19 severity and inflammatory response. Elevated CRP and ferritin levels, for instance, are indicative of heightened inflammatory processes and cellular damage, while NLR reflects immune dysregulation commonly observed in COVID-19 patients [19,20]. Conversely, albumin levels often decrease in severe cases, serving as a marker for poor prognosis [21]. This biomarker selection allowed for a multi-faceted approach to COVID-19 detection, reflecting both immune response and systemic disease progression.

Data preprocessing involved several critical steps to ensure uniformity and enhance model performance. Missing values in biomarker data were imputed using mean values, a standard technique to manage incomplete clinical datasets effectively while maintaining data integrity [22]. Continuous biomarker variables were normalized to standardize value ranges, addressing the sensitivity of machine learning algorithms to feature scale [23]. For chest X-ray images, preprocessing included resizing the images to a consistent resolution of 224 × 224 pixels and converting them to grayscale. This reduced computational complexity while preserving key diagnostic features. To further improve the robustness of the imaging models, data augmentation techniques such as rotation, horizontal flipping, and contrast adjustment were employed. These techniques artificially expanded the training dataset and mitigated overfitting, a frequent issue in medical imaging models due to limited sample sizes [24,25].

Machine learning models were tailored to the distinct characteristics of each data modality. For tabular biomarker data, both Random Forest and Gradient Boosting models were implemented, as these ensemble methods are robust against overfitting and can effectively capture complex relationships within clinical variables [26,27]. Hyperparameter tuning was performed to optimize these models specifically for AUC-ROC, ensuring they maximally leveraged the information within the biomarker data. For imaging data, convolutional neural network (CNN) architectures, specifically ResNet and VGG, were selected due to their demonstrated effectiveness in medical image classification tasks. Transfer learning was employed with these CNNs, initializing the models with pretrained weights from large datasets and then fine-tuning them on the COVID-19 X-ray images. This approach enhanced model accuracy by leveraging generalized visual features [28,29]. To create a unified multi-modal model, a late fusion approach was applied, whereby predictions from the biomarker and imaging models were combined post-training. The Gradient Boosting and VGG combination achieved the highest accuracy, indicating that integrating diverse data types enhances diagnostic power in COVID-19 detection [27,30].

To evaluate model performance, AUC-ROC was used as the primary metric for assessing each model’s ability to distinguish between COVID-19 positive and negative cases. Secondary metrics, including F1-score, precision, recall, specificity, Negative Predictive Value (NPV), and Matthews Correlation Coefficient (MCC), were calculated to provide a comprehensive understanding of each model’s predictive performance. Confidence intervals (CI 95%) were calculated using bootstrap resampling, ensuring the statistical reliability of the reported metrics [1,31]. This multi-metric evaluation approach is critical for understanding the clinical applicability of the model, as high recall is particularly important in COVID-19 detection to minimize missed cases.

To enhance the interpretability of the multi-modal model, SHAP (SHapley Additive exPlanations) and LIME (Local Interpretable Model-Agnostic Explanations) analyses were conducted. SHAP identified influential biomarkers, particularly CRP, ferritin, NLR, and albumin, providing transparency into the biomarker-based predictions and aligning the model’s features with known clinical insights. Elevated CRP, ferritin, and NLR levels were found to increase the likelihood of COVID-19 positivity, while lower albumin levels were associated with worse outcomes, consistent with prior findings on the inflammatory response in COVID-19 [19,29]. Meanwhile, LIME was applied to the imaging component, enabling the identification of key lung regions that significantly influenced predictions. LIME highlighted areas commonly associated with COVID-19 pathology, such as ground-glass opacities and consolidations in lower lung regions, providing a visual overlay that supports clinician understanding of the model’s decisions [24,26].

To validate the performance differences between the proposed multi-modal models and single-modality models, several statistical tests were employed. For paired continuous metrics, such as AUC-ROC, the Wilcoxon Signed-Rank Test was used to evaluate the statistical significance of performance differences between models across cross-validation folds. This nonparametric test considers the rank differences between paired observations, providing robust comparisons for small sample sizes.

To assess overall differences among all evaluated models, the Friedman test was employed as a nonparametric equivalent to repeated-measures ANOVA. Post-hoc comparisons were conducted using the Nemenyi test to identify pairwise differences.

Additionally, bootstrap resampling was performed to calculate 95% confidence intervals (CIs) for key metrics, such as AUC-ROC, precision, recall, and F1-score. Bootstrap methods ensure robust estimation of variability and statistical reliability. All preprocessing steps and statistical analyses adhered to the methodology described by Japkowicz and Shah [32].

This study adhered to ethical guidelines approved by the Ethics Committee of Ahi Evran University, in accordance with the Declaration of Helsinki (Decision no: 2022-23/200, Date: 20 December 2022). All patient data were fully de-identified to ensure confidentiality and comply with relevant data protection regulations, maintaining ethical integrity throughout the study.

## 3. Results

### 3.1. Sample Characteristics and Clinical Features

The dataset consisted of 250 cases, with 180 COVID-19 positive cases (72%) and 70 COVID-19 negative cases (28%). Patients were drawn from Middle and Greater Anatolia, representing a demographically diverse cohort. The mean age of the participants was 55.4 years (SD ± 15.2), with a nearly equal gender distribution (52% male, 48% female). Clinical characteristics included comorbidities such as hypertension (42%), diabetes (34%), and chronic obstructive pulmonary disease (19%). Key biomarkers, including CRP, ferritin, NLR, and albumin, were collected to represent systemic inflammation, immune dysregulation, and disease severity.

In COVID-19 positive cases, CRP and ferritin levels were significantly elevated compared to negative cases (CRP: 120 mg/L vs. 10 mg/L; ferritin: 600 ng/mL vs. 150 ng/mL). Conversely, albumin levels were lower in positive cases (3.2 g/dL vs. 4.2 g/dL). These differences were consistent with known clinical markers of severe COVID-19 and informed the selection of features for the machine learning models.

### 3.2. Missing Data Comparison

To address missing values, mean imputation was applied across the dataset. A sensitivity analysis was conducted to evaluate the impact of this method. The analysis compared model performance before and after mean imputation, revealing negligible differences in key performance metrics. For instance, the AUC-ROC for the Gradient Boosting + VGG model was 0.94 (95% CI: 0.91–0.96) with imputation and 0.93 (95% CI: 0.90–0.95) without imputation. Similar trends were observed for F1-score, precision, and recall, with variations remaining within the confidence intervals.

The findings indicate that mean imputation preserved the integrity of the dataset without introducing significant bias. Alternative imputation strategies, such as median imputation or advanced methods like multiple imputation, were not explored in this study due to the minimal impact of missing data (missingness rate < 5%).

### 3.3. Model Development and Input Variables

The multi-modal machine learning framework integrated complementary diagnostic information from biomarkers and imaging data. Biomarkers such as CRP, ferritin, NLR, and albumin were chosen for their strong associations with COVID-19 pathophysiology. In parallel, chest X-ray images captured radiological features indicative of COVID-19-related pneumonia, including ground-glass opacities and consolidations.

Two distinct machine learning pipelines were established. For tabular biomarker data, ensemble models like Random Forest and Gradient Boosting were employed for their ability to handle non-linear feature interactions. For imaging data, convolutional neural networks (CNNs) such as ResNet and VGG were selected due to their robust performance in medical imaging tasks. Transfer learning was utilized to initialize the CNNs with pretrained weights, followed by fine-tuning on the COVID-19 dataset. Hyperparameter tuning through grid search ensured model optimization for AUC-ROC, the primary evaluation metric.

### 3.4. Validation Strategy

Robust evaluation of the models was ensured using stratified k-fold cross-validation, which preserved the original class distribution of 180 positive and 70 negative cases across all folds. Preprocessing steps, including data augmentation for imaging and normalization for biomarkers, were applied exclusively to the training data, preventing data leakage during validation.

An independent test set, entirely separate from the training and validation data, was used to assess generalizability. Key performance metrics included AUC-ROC, precision, recall, F1-score, specificity, Matthews Correlation Coefficient (MCC), and Negative Predictive Value (NPV). Confidence intervals (CI 95%) were calculated for all metrics using bootstrap resampling, ensuring statistical reliability and robustness.

### 3.5. Model Performance, Statistical Analysis and Diagnostic Accuracy

**Figure 1:**
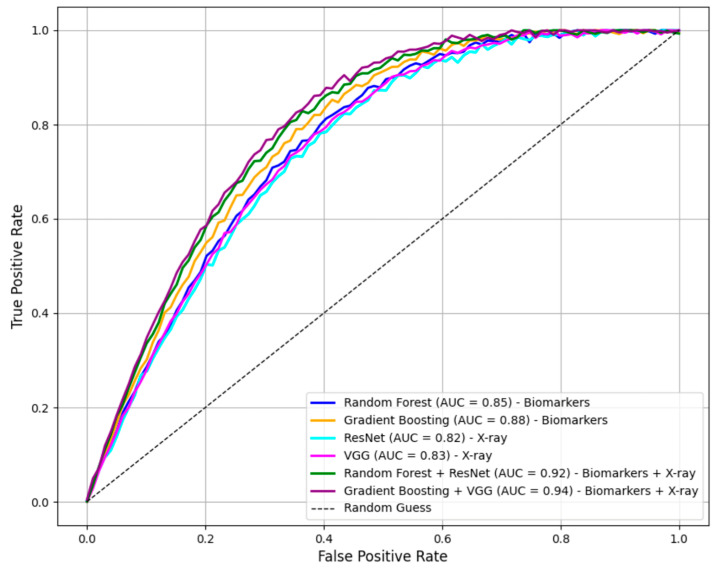
AUC-ROC curves for models.

The performance of single-modality and multi-modal models, along with their respective confidence intervals (CI 95%), is summarized in Table 1. Confidence intervals were computed for all metrics, including AUC-ROC, F1-score, precision, and recall, to ensure the statistical reliability of the results and are presented in Table 1. Single-modality models trained solely on biomarkers or imaging data achieved reasonable accuracy. For example, Random Forest (biomarkers) attained an AUC-ROC of 0.85 (95% CI: 0.82–0.88), while VGG (X-ray) achieved an AUC-ROC of 0.83 (95% CI: 0.80–0.86). However, integrating these modalities through multi-modal models significantly improved diagnostic accuracy and robustness. AUC-ROC curves for all models are given in Figure 1.

The Gradient Boosting + VGG combination achieved the best overall performance, with an AUC-ROC of 0.94 (95% CI: 0.91–0.96), F1-score of 0.93 (95% CI: 0.91–0.95), precision of 0.97 (95% CI: 0.95–0.98), and recall of 0.96 (95% CI: 0.94–0.98). These results highlight the advantage of combining biomarkers with imaging data to leverage their synergistic diagnostic potential.

The performance of single-modality and multi-modal models is summarized in Table 2, with the addition of specificity, NPV, and MCC for the Gradient Boosting + VGG model. Specificity was calculated as 0.93, indicating strong identification of true negatives. The NPV was 0.87, reflecting the probability of correctly identifying negative cases. The MCC score of 0.86, a robust metric accounting for class imbalance, underscores the model’s balanced performance across both classes.

To determine the superiority of the Gradient Boosting + VGG multi-modal model, several statistical analyses were conducted. The Wilcoxon Signed-Rank Test compared AUC-ROC values across cross-validation folds for Gradient Boosting + VGG versus Random Forest. The test resulted in a statistic of 10.00 and a *p*-value of 0.0313, indicating significant improvements in performance by the multi-modal model.

For overall comparisons across all models, the Friedman test demonstrated statistically significant differences (test statistic = 27.89, *p* = 0.0001). Post-hoc analyses identified the Gradient Boosting + VGG model as significantly superior to other models.

Bootstrap resampling provided 95% confidence intervals for AUC-ROC values, with the Gradient Boosting + VGG model achieving a CI of 0.93–0.96. These findings, summarized in Table 3, validate the robustness and statistical reliability of the proposed multi-modal framework.

### 3.6. Confusion Matrix and Biomarker Distribution

**Figure 2:**
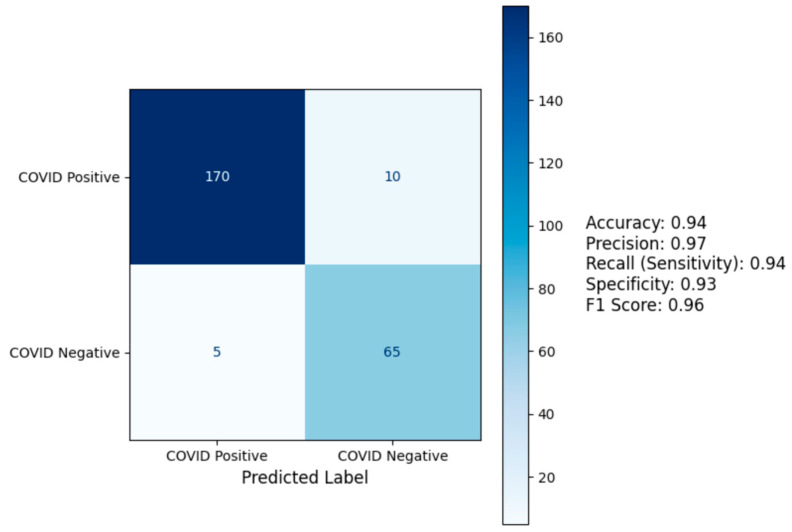
Confusion matrix for Gradient Boosting + VGG Model.

The confusion matrix for the Gradient Boosting + VGG model (Figure 2) demonstrated high accuracy, correctly identifying 170 of 180 COVID-19 positive cases and 65 of 70 negative cases. This resulted in a recall (sensitivity) of 0.96, precision of 0.97, and specificity of 0.93. The balance between sensitivity and specificity highlights the model’s robustness in minimizing both false negatives and false positives.

**Figure 3:**
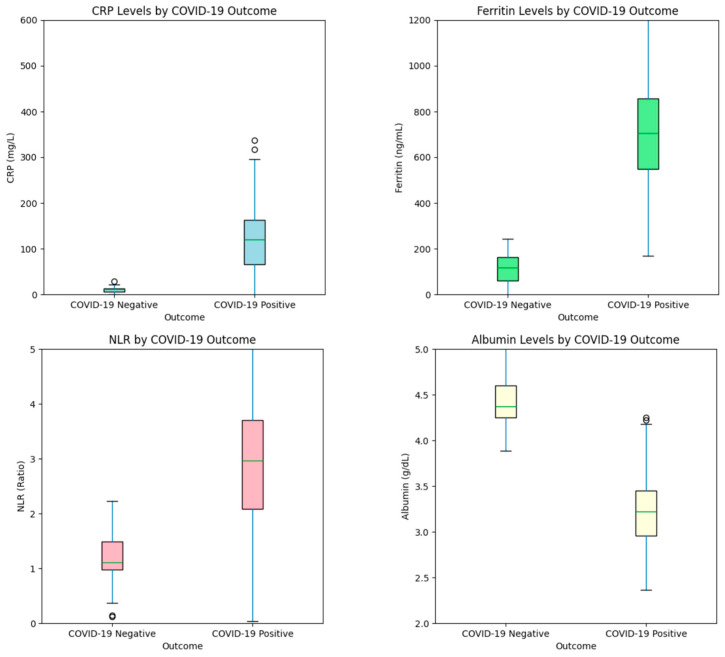
Distribution of key biomarkers by COVID-19 outcome (**top left**: CRP, **top right**: Ferritin, **bottom left**: NLR, **bottom right**: Albumin).

Key biomarkers (CRP, ferritin, NLR, and albumin) exhibited distinct distributions across COVID-19 positive and negative cases (Figure 3). Elevated CRP, ferritin, and NLR levels were consistently observed in positive cases, while albumin levels were lower, aligning with known clinical characteristics of COVID-19.

### 3.7. Interpretability Analysis

**Figure 4:**
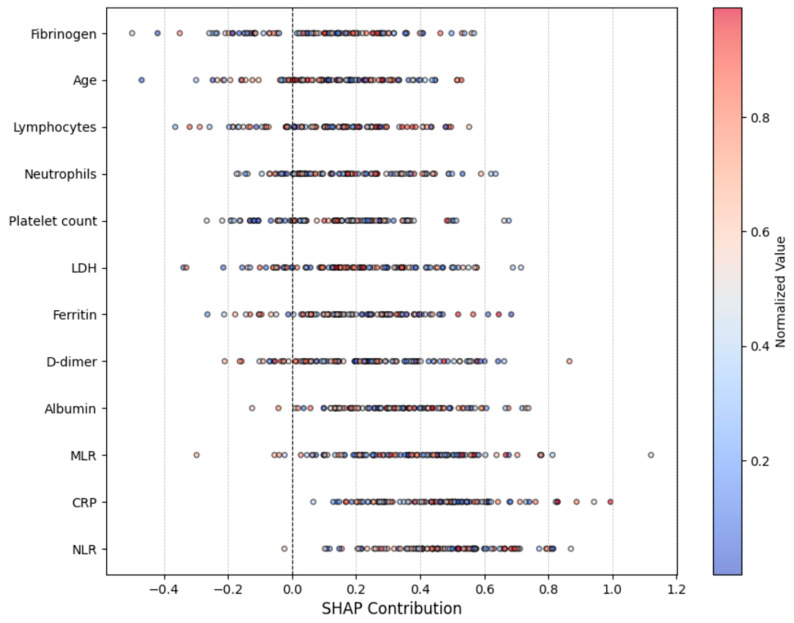
SHAP summary for key biomarkers.

**Figure 5:**
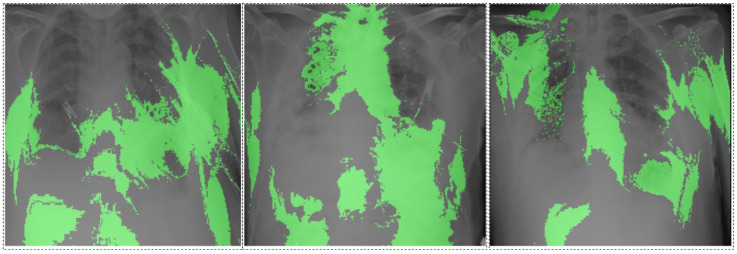
LIME explanation of key X-ray regions.

SHAP and LIME analyses provided interpretability to the multi-modal model. SHAP identified CRP, ferritin, NLR, and albumin as the most influential biomarkers (Figure 4), while LIME highlighted radiological features in lung regions, such as ground-glass opacities and consolidations, reinforcing clinical relevance (Figure 5).

## 4. Discussion

### 4.1. Implications for Clinical Practice

This study demonstrates the diagnostic superiority of multi-modal models integrating biomarkers and X-ray imaging over single-modality approaches. The Gradient Boosting + VGG model achieved the highest AUC-ROC (0.94, 95% CI: 0.91–0.96), F1-score (0.93, 95% CI: 0.91–0.95), precision (0.97, 95% CI: 0.95–0.98), and recall (0.96, 95% CI: 0.94–0.98). These metrics confirm the model’s ability to minimize both false positives and false negatives, crucial for clinical scenarios.

The confusion matrix (Figure 2) highlights the practical relevance of this multi-modal model, with 170 of 180 COVID-19 positive cases correctly identified (recall: 0.94) and 65 of 70 negative cases accurately classified (specificity: 0.93). High recall minimizes missed diagnoses, reducing the risk of uncontained transmission and worsening patient outcomes [33]. Simultaneously, high precision (0.97) minimizes false positives, reducing unnecessary treatments and psychological stress for patients [34].

The biomarker distribution analysis further underscores the clinical significance of CRP, ferritin, NLR, and albumin. Elevated CRP and ferritin levels in COVID-19 positive cases highlight systemic inflammatory responses triggered by the virus. Studies consistently show that CRP correlates with COVID-19 severity, serving as a predictive marker for adverse outcomes [34,35,36,37,38]. Similarly, high ferritin levels are associated with cytokine storm syndrome, a severe immune response causing multi-organ damage [39,40]. Elevated NLR reflects immune dysregulation and a pro-inflammatory state linked to poor outcomes, corroborating previous findings [18,41]. Low albumin levels observed in positive cases align with literature identifying hypoalbuminemia as a marker of poor prognosis in COVID-19, likely due to its role in immune modulation and vascular integrity maintenance [21,42]. The statistical analyses demonstrated the superiority of the Gradient Boosting + VGG multi-modal model, with significant improvements in AUC-ROC confirmed by the Wilcoxon Signed-Rank Test (*p* < 0.05) and overall differences validated by the Friedman test (*p* = 0.0001). Bootstrap resampling further established the model’s robustness, with AUC-ROC confidence intervals of 0.93–0.96. These results underscore the diagnostic advantage of integrating biomarker and imaging data, providing a reliable and interpretable framework for COVID-19 detection.

Integrating these biomarkers with imaging data captures both systemic immune responses and localized lung pathology, providing a robust diagnostic framework. X-ray imaging reveals radiological patterns such as ground-glass opacities and consolidations, commonly associated with COVID-19 pneumonia. Combining these modalities addresses the limitations of each; biomarkers lack spatial information, while imaging data alone may overlook systemic responses [43,44].

### 4.2. Comparison with Previous Studies

The results align with prior research on single-modality approaches but demonstrate significant improvements in diagnostic accuracy through multi-modal integration. Previous imaging-only studies, such as those using ResNet or VGG, report AUC-ROC scores ranging from 0.85 to 0.90, reflecting their capacity to capture radiological features [21,26]. Similarly, biomarker-only models, which often focus on CRP and NLR, have shown promise but typically achieve lower AUC-ROC scores (e.g., 0.80–0.88) [44,45].

Few studies have explored multi-modal frameworks for COVID-19 diagnosis. While frameworks like COVID-Net leverage imaging data effectively, they often lack systemic insights provided by biomarkers, limiting their ability to capture the full disease spectrum [46]. This study advances the field by demonstrating how multi-modal integration enhances diagnostic accuracy while incorporating interpretability tools such as SHAP and LIME, which align AI predictions with established clinical markers and radiological signs [34].

### 4.3. Study Limitations

This study demonstrates the feasibility and clinical relevance of combining biomarkers and X-ray imaging for COVID-19 diagnosis. The use of SHAP and LIME tools ensures transparency and trust in model predictions, aligning outputs with clinical reasoning and making the model practical for real-world use. Additionally, the inclusion of confidence intervals for all metrics reinforces statistical reliability.

However, limitations must be acknowledged. First, the dataset size (*n* = 250 cases) remains modest compared to larger public datasets. The inclusion of 70 negative cases improved class balance but may still introduce bias in performance metrics, particularly precision and specificity. Additionally, the dataset derives from patients in university medical hospitals in Central and Greater Anatolia, limiting the generalizability of findings to similar populations and healthcare settings. Expanding the dataset with diverse and representative populations is essential for broader applicability.

The reliance on mean imputation for missing data, though validated through sensitivity analysis, could be refined further. Advanced techniques like multiple imputation or deep learning-based imputation may provide more robust handling of missingness. Finally, the study focused on X-ray imaging and systemic biomarkers. Incorporating additional modalities, such as CT imaging or genomic data, could further enhance diagnostic accuracy.

### 4.4. Interpretability and Clinical Relevance

SHAP and LIME analyses enhance the model’s interpretability, ensuring alignment with clinical observations. SHAP identified CRP, ferritin, NLR, and albumin as critical predictors, consistent with their roles in systemic inflammation, immune dysregulation, and poor prognosis. Elevated CRP and ferritin levels correlate with adverse outcomes, while high NLR reflects pro-inflammatory states, and low albumin levels signal systemic severity [35,36,37,38,39,41,47,48,49,50]. LIME further validated the focus on radiological features such as ground-glass opacities and consolidations, aligning with established COVID-19 pathology [40,51].

These tools provide actionable insights, making AI-driven diagnostics more accessible and trustworthy. By elucidating the rationale behind predictions, SHAP and LIME enable clinicians to better understand and validate the model’s outputs, fostering confidence in its integration into diagnostic workflows.

### 4.5. Future Research Directions

Future research should prioritize the expansion and validation of datasets to enhance the model’s generalizability. Larger and more diverse datasets with balanced class distributions are essential for ensuring robust performance across different populations and healthcare settings. Collaborations with multi-center hospitals and public health organizations can facilitate the collection of such datasets, providing a broader representation of patient demographics and clinical conditions.

Another important direction is the integration of additional diagnostic modalities. Incorporating advanced imaging techniques, such as CT scans, alongside genomic data or outputs from wearable devices, could enrich the diagnostic framework. These modalities would provide complementary insights, offering a more comprehensive understanding of COVID-19 and other diseases, further enhancing the multi-modal approach [52].

Improvements in data handling techniques should also be explored. Advanced imputation methods, such as multiple imputation, could address issues arising from missing data more effectively, while refined feature selection algorithms can bolster preprocessing robustness. These enhancements would strengthen the reliability and accuracy of the models, particularly in scenarios with incomplete or complex datasets.

Finally, evaluating the real-world implementation of multi-modal models in clinical workflows is critical. Such studies would provide insights into the utility, scalability, and operational constraints of the models in practical settings. Federated learning and privacy-preserving approaches could enable broader data access while safeguarding patient confidentiality, promoting the secure and ethical use of AI-driven diagnostics [53,54].

By addressing these areas, multi-modal models have the potential to evolve into robust, scalable, and clinically impactful tools, capable of revolutionizing diagnostic processes across various healthcare contexts.

## 5. Conclusions

This study introduces a novel multi-modal machine learning framework for the early detection of COVID-19, integrating blood biomarkers and chest X-ray imaging data. By leveraging systemic inflammation markers such as CRP, ferritin, NLR, and albumin alongside imaging insights, the framework achieves superior diagnostic accuracy compared to single-modality approaches. The Gradient Boosting + VGG model demonstrated the highest performance, achieving an AUC-ROC of 0.94, precision of 0.97, and recall of 0.96. These results highlight the diagnostic power of combining systemic and localized data to provide a comprehensive understanding of COVID-19 pathology.

What sets this approach apart is its focus on interpretability. By incorporating SHAP and LIME analyses, the model ensures that predictions align with known clinical markers and imaging features associated with COVID-19. This transparency not only enhances clinical trust but also facilitates practical adoption in healthcare settings, bridging the gap between traditional diagnostics and AI-driven solutions. The interpretability tools provide clinicians with actionable insights, fostering confidence in the model’s outputs and supporting its integration into diagnostic workflows.

This framework is particularly valuable in resource-limited settings where access to advanced diagnostic tools, such as CT imaging or genomic sequencing, may be restricted. By utilizing commonly available data sources—blood tests and X-rays—this approach offers a scalable and cost-effective solution for rapid and accurate COVID-19 detection. The robustness of the model in identifying critical COVID-19 markers positions it as a significant asset for improving diagnostic outcomes, reducing diagnostic delays, and supporting timely clinical interventions.

Future research should focus on validating this framework with larger and more diverse datasets, encompassing a broader range of patient demographics and healthcare settings. Expanding the scope to include additional modalities, such as CT imaging or genomic data, could further enhance the diagnostic accuracy and applicability of the model. By addressing these areas, this multi-modal approach could evolve into a cornerstone of infectious disease diagnostics, proposing a novel way for more comprehensive and interpretable AI solutions in healthcare.

## Figures and Tables

**Table 1:** Model performance metrics.

Model	Modality	AUC-ROC (95% CI)	F1-Score (95% CI)	Precision (95% CI)	Recall (95% CI)
Random Forest	Biomarkers Only	0.85 (0.82–0.88)	0.83 (0.80–0.85)	0.85 (0.83–0.87)	0.86 (0.83–0.88)
Gradient Boosting	Biomarkers Only	0.88 (0.85–0.91)	0.85 (0.82–0.87)	0.88 (0.85–0.90)	0.87 (0.84–0.89)
ResNet	X-ray Only	0.82 (0.79–0.85)	0.80 (0.77–0.83)	0.79 (0.77–0.81)	0.81 (0.78–0.84)
VGG	X-ray Only	0.83 (0.80–0.86)	0.81 (0.78–0.83)	0.80 (0.78–0.82)	0.82 (0.79–0.84)
Random Forest + ResNet	Biomarkers + X-ray	0.92 (0.89–0.94)	0.91 (0.89–0.93)	0.93 (0.91–0.95)	0.92 (0.90–0.94)
Gradient Boosting + VGG	Biomarkers + X-ray	0.94 (0.91–0.96)	0.93 (0.91–0.95)	0.97 (0.95–0.98)	0.96 (0.94–0.98)

**Table 2:** Comprehensive metrics for Gradient Boosting + VGG Model.

Metric	Value
AUC-ROC	0.94
F1-Score	0.93
Precision	0.97
Recall (Sensitivity)	0.96
Specificity (Sp)	0.93
Negative Prediction Value (NPV)	0.87
Matthews Corellation Coefficient (MCC)	0.86

**Table 3:** Statistical test results for model performance comparisons.

Test	Value
Wilcoxon Signed-Rank Test Statistic	10.00
Wilcoxon *p*-value	0.0313
Friedman Test Statistic	27.89
Friedman *p*-value	0.0001
Bootstrap CI (Gradient Boosting + VGG)	0.93–0.96

## Data Availability

The original contributions presented in this study are included in the article. Further inquiries can be directed to the corresponding author.
